# Interactive Effects of Light and Melatonin on Biosynthesis of Silymarin and Anti-Inflammatory Potential in Callus Cultures of *Silybum marianum* (L.) Gaertn.

**DOI:** 10.3390/molecules24071207

**Published:** 2019-03-27

**Authors:** Muzamil Shah, Muhammad Asad Ullah, Samantha Drouet, Muhammad Younas, Duangjai Tungmunnithum, Nathalie Giglioli-Guivarc’h, Christophe Hano, Bilal Haider Abbasi

**Affiliations:** 1 Department of Biotechnology, Quaid-i-Azam University, Islamabad-45320, Pakistan; muzamilshah1989@gmail.com (M.S.); asad_ullah8050@yahoo.com (M.A.U.); pk.younas@gmail.com (M.Y.); 2 Laboratoire de Biologie des Ligneux et des Grandes Cultures (LBLGC), INRA USC1328, Université d’Orléans, 45067 Orléans CEDEX 2, France; samantha.drouet@univ-orleans.fr (S.D.); duangjai.tun@mahidol.ac.th (D.T.); 3 COSM’ACTIFS, Bioactifs et Cosmétiques, CNRS GDR3711, 45067 Orléans CEDEX 2, France; 4 Department of Pharmaceutical Botany, Faculty of Pharmacy, Mahidol University, 447 Sri-Ayuthaya Road, Rajathevi, Bangkok 10400, Thailand; 5 EA2106 Biomolecules et Biotechnologies Vegetales, Universite Francois-Rabelais de Tours, 37000 Tours, France; nathalie.guivarch@univ-tours.fr

**Keywords:** *Silybum marianum* (L.) Gaertn., light regimes, melatonin, antioxidant, phenolics, flavonoids, silymarin, anti-inflammatory

## Abstract

*Silybum marianum* (L.) Gaertn. is a well-known medicinal herb, primarily used in liver protection. Light strongly affects several physiological processes along with secondary metabolites biosynthesis in plants. Herein, *S. marianum* was exploited for in vitro potential under different light regimes in the presence of melatonin. The optimal callogenic response occurred in the combination of 1.0 mg/L α-naphthalene acetic acid and 0.5 mg/L 6-benzylaminopurine under photoperiod. Continuous light associated with melatonin treatment increased total flavonoid content (TFC), total phenolic content (TPC) and antioxidant potential, followed by photoperiod and dark treatments. The increased level of melatonin has a synergistic effect on biomass accumulation under continuous light and photoperiod, while an adverse effect was observed under dark conditions. More detailed phytochemical analysis showed maximum total silymarin content (11.92 mg/g dry weight (DW)) when placed under continuous light + 1.0 mg/L melatonin. Individually, the level of silybins (A and B), silydianin, isolsilychristin and silychristin was found highest under continuous light. Anti-inflammatory activities were also studied and highest percent inhibition was recorded against 15-lipoxygenase (15-LOX) for cultures cultivated under continuous light (42.33%). The current study helps us to better understand the influence of melatonin and different light regimes on silymarin production as well as antioxidant and anti-inflammatory activities in *S. marianum* callus extracts.

## 1. Introduction

*Silybum marianum* (L.) Gaertn. (Family *Asteraceae*), commonly called milk thistle, is an important medicinal herb with a potent hepatoprotective activity [1]. The annual average sale of *S. marianum* is about 8 billion USD and the demand per year varies from 18 to 20 tons [2]. Silymarin is the prominent component in *S. marianum*, which is an isomeric mixture of several compounds including the flavonolignans silybins, silychristin, isosilybins, and silydianin associated with the flavonoid taxifolin. Due to its well described free radical scavenging capacity, silymarin can protect human hepatic tissues by neutralizing the effect of oxidative damage [3]. Studies from in vivo and in vitro animal models suggest the protective role of silymarin on hepatic cells from toxin [4]. A variety of active ingredients are produced in plants during development in which phenolic compounds have a distinct identity as antioxidant agents [5]. Redox properties of the compounds are generally responsible for antioxidant activity [6], which enable them to act as hydrogen-atom donors or reducing agents [7]. Silymarin is known to exhibit various medicinal properties including antiviral, anti-diabetic, anticancer, anti-inflammatory, anti-arthritic, antioxidant and immunomodulatory [2,8,9,10,11,12]. Additionally, silymarin extract has also been shown to be effective in treating non-alcoholic fatty liver disorder (NAFLD), obsessive-compulsive disorder (OCD) and β-thalassemia [13,14,15]. Anti-inflammatory activities of plant extracts are usually assessed by measuring % inhibition of COX-1 (cyclooxygenase 1), COX-2 (cyclooxygenase 2), sPLA2 (secretory phospholipase A2) and 15-LOX (15-lipoxygenase). Cyclooxygenases (COXs) are endogenous enzymes that help to maintain tissue homeostasis of kidney, platelets, gastrointestinal tract and expressed in different types of cancers [16]. COXs are the key player in inflammation process and are usually the main target for development of NSAIDs (non-steroidal anti-inflammatory drugs). COX-1 is known as a house-keeping enzyme, whereas COX-2 produces prostaglandin E2 which is an endogenous pain-producing molecule. Some anti-inflammatory drugs consists of the molecular mechanism that inhibit both COX-2 and COX-1 enzymes. So, these drugs which inhibit both COX-1 and COX-2 enzymes can cause undesirable side effects such as renal dysfunction and/or gastrointestinal bleeding. Thus, researchers nowadays seek better candidates that can inhibit only COX-2 expression for drug discovery and development [17]. The current review on chemistry, pharmacological activities and nutraceutical uses of *S. marianum* in liver diseases reported that silymarin, a major phytochemical compound from *S. marianum*, exhibited both anti-inflammatory and immunomodulatory potentials by inhibiting NF-κB pathway [1]. Moreover, silymarin and the *S. marianum* preparations show a low level of drug interaction [1]. Interestingly, the review on the effect of *S. marianum* in metabolic syndrome on both animal models and clinical trials conducted on humans suggested that silymarin has potential to be an alternative choice for treatment of metabolic syndrome disease [18]. Plants are primary source of compounds that inhibit these key enzymes during inflammation process by acting as natural inhibitors [19,20]. Multiple biotic as well as abiotic elicitors have previously been employed in vitro in several medicinal plant species to increase the content of secondary metabolites. Elicitors alter plant metabolism by provoking physiological cascades which leads to enhanced biosynthesis of phytochemicals [21,22]. As a chief abiotic elicitor, light affects various physiological processes (such as, photosynthesis), hence morphogenesis, development and growth of several medicinal plants in vitro [23,24].

Melatonin (*N*-acetyl-5-methoxytryptamine), discovered initially in vertebrates, is a naturally occurring indole amine [25], which, after its detection as phytohormone, has also been spotted in various plant species (oats, rice, corn, barley and wheat) [26,27,28,29]. Manchester et al. [29] reported higher concentration of melatonin in white and black seeds of mustard as compared to the level in the blood of vertebrates. Melatonin higher levels comparatively in plants are assumed to be plants compensatory response as, unlike animals, they are devoid of mobility to cope with extreme environments. Numerous reports indicate the role of melatonin in root development, division of cells [30], photoperiod dependent processes and regulation of circadian rhythms [31,32,33]. Moreover, due to similarities in structure, it has also been used as a substitute to IAA (Indole-3-acetic acid) [34]. Photoperiod influences the endogenous melatonin level and elevated level of melatonin have been detected during the dark in plants [35]. The current study was designed to explore the interactive effect of light and melatonin on silymarin biosynthesis in *S. marianum* callus cultures and its antioxidant and anti-inflammatory potential.

## 2. Results and Discussion

### 2.1. Interactive Effect of Light and Melatonin on Biomass Accumulation 

**Figure 1:**
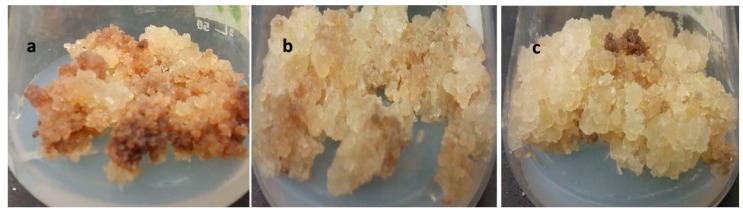
Effect of light and melatonin on *S. marianum* callus morphology. (**a**) Dark + 0.5 mg/L melatonin. (**b**) Photoperiod + 0.5 mg/L melatonin (**c**) Continuous light + 1.0 mg/L melatonin.

Plants have the capacity to perceive and process information for optimum growth and development from their biotic and abiotic environments [36]. As an abiotic elicitor, light has a vital role in a plant’s growth and development by regulating indigenous metabolic activities and maintaining hormonal balance [35]. On the other hand, melatonin is known to provide defense against biotic and abiotic stressors [37,38]. The current study investigated the effect of melatonin on callus cultures of *S. marianum* placed under different light regimes. Application of different melatonin concentrations under three light regimes (dark (24 h), photoperiod (16 h/8 h) and continuous white light (24 h)) was studied for optimal biomass production. Highest biomass production (15.9 g/L dry weight (DW)) occurred in callus culture treated with 1.0 mg/L melatonin grown under continuous white light (Table 1), while lowest biomass accumulation (7.54 g/L) was noted in the dark grown cultures with 10.0 mg/L melatonin. Under the photoperiod cycle, highest biomass accumulation (14.37 mg/L) happened at 0.5 mg/L melatonin (Figure 1). Overall, continuous light and photoperiod showed a profound effect with moderate melatonin concentrations, whereas an increase in melatonin concentration exhibited inhibitory effects on biomass accumulation in the dark grown cultures. Similarly, study conducted by Fazal et al. [39] indicated that moderate concentration of exogenous melatonin treatment produces optimum results in callus culture of *P. vulgaris*. Khan et al. [40] also studied the interrelating effect of light and melatonin in callus cultures of *Fagonia indica* for enhanced production of anticancer compounds. The results of their study suggested that continuous white light with melatonin is most effective as compared to other light treatments. Adil et al. [41] also concluded that combined treatment of melatonin and continuous light display optimum biomass and secondary metabolites accumulation in adventitious roots culture of *W. somnifera*. 

### 2.2. Interactive Effect of Light and Melatonin on Accumulation of Secondary Metabolites 

**Figure 2:**
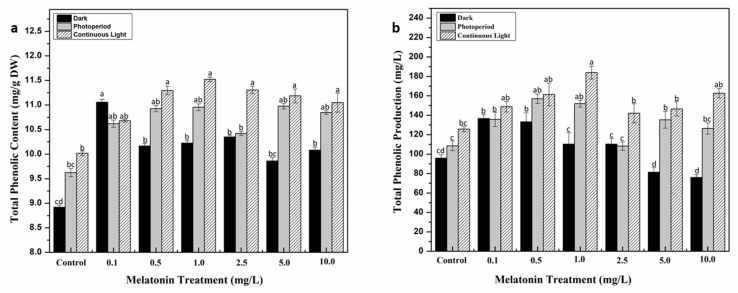
Phenolic accumulation in response to different melatonin and lights treatments. (**a**) Total phenolic content. (**b**) Total phenolic production. Values are means ± SD from three replicates. Columns with similar alphabets are not significantly different (*p* < 0.05).

**Figure 3:**
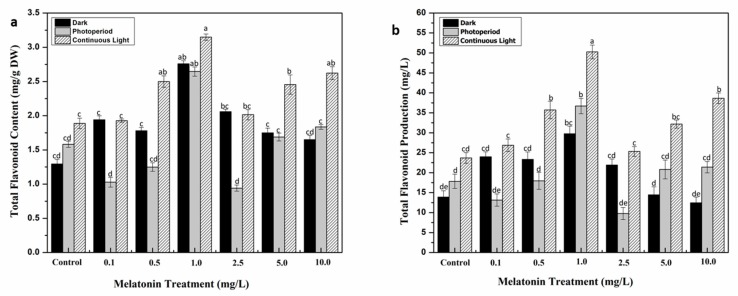
Flavonoids accumulation in response to different melatonin and lights treatments. (**a**) Total flavonoid content. (**b**) Total flavonoid production. Values are means ± SD from three replicates. Columns with similar alphabets are not significantly different (*p* < 0.05).

Plants have indigenous defense mechanism comprising large array of molecules that help them to survive and grow in response to a variety of environmental conditions including biotic and abiotic stresses. The major constituents of these phytochemicals are phenolics and flavonoids, which are released in unfavorable conditions [42,43]. In this study, the influence of melatonin and different light regimes on biosynthesis of these metabolites was also investigated. Callus cultures supplemented with various concentrations of melatonin were grown in three different light regimes. The highest total phenolic and flavonoid contents (TPC and TFC, respectively) were documented in callus cultures grown under continuous white light for all melatonin concentrations as compared to the rest of light regimes. Among all the treatments, 1.0 mg/L of melatonin showed highest TPC (11.522 mg/g) and TFC (3.149 mg/g) under continuous light, followed by TPC (11.3 mg/g) and TFC (2.49 mg/g) at 0.5 mg/L melatonin concentration (Figure 2a and Figure 3a). As for the dark treated cultures, the inverse relation of melatonin concentration and secondary metabolites production was observed i.e., metabolites accumulation was retarded with increase in melatonin concentration. Total phenolic and flavonoid productions (TPP and TFP, respectively) were also estimated by multiplying the TPC and TFC values of the cultures with their respective dry weights. Similar trend of highest TPP (183.84 mg/L) and TFP (50.25 mg/L) was recorded in cultures grown under continuous light with 1.0 mg/L melatonin (Figure 2b and Figure 3b). A positive correlation in biomass production and metabolites accumulation was observed in this study. Melatonin plays a contributing role in defense initiation in plants under stress conditions by regulating gene expression machinery which favors biosynthesis of specialized metabolites [44], whereas, light is regarded as an effective abiotic stress inducer in plants [45,46]. An optimal level of melatonin in response to light stress could be the only reason behind enhanced metabolites accumulation in callus culture of *S. marianum*. The interrelating effect of melatonin and light has recently been studied by Khan et al. [40] in callus cultures of *F. indica*, revealing a maximum production of secondary metabolites at moderate melatonin concentration (10 µM) under continuous light. Similarly, Adil et al. [41] also concluded the synergistic effect of melatonin and light on enhanced synthesis of bioactive ingredients in *W. somnifera* adventitious roots culture. The influence of light on in vitro derived cultures has previously been studied in several other plant species with respect to their phytochemical production [47,48].

### 2.3. Effect of Light and Melatonin on Antioxidant Activities 

**Figure 4:**
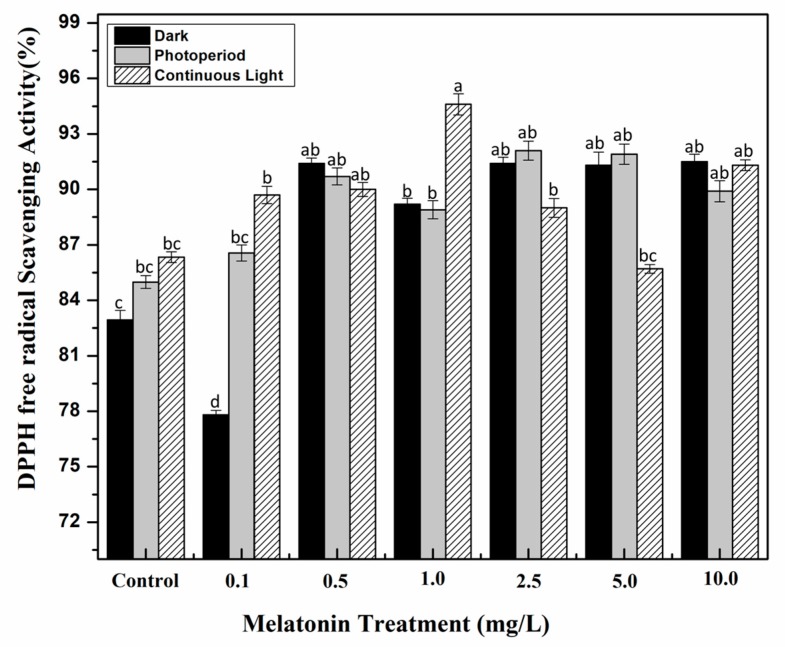
In vitro DPPH (2,2-diphenyl-1-picrylhydrazyl) antioxidant activity of *S. marianum* calli grown under different light regimes and melatonin treatments. Values are means ± SD from three replicates. Columns with similar alphabets are not significantly different (*p* < 0.05).

**Figure 5:**
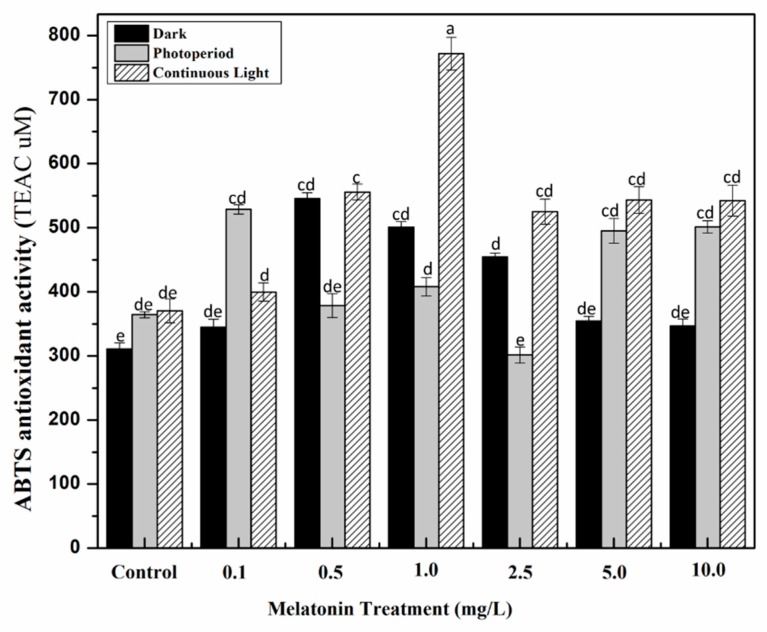
In vitro ABTS (2,2-azinobis-3-ethylbenzthiazoline-6-sulphonic acid) antioxidant activity of *S. marianum* calli grown under different light regimes and melatonin treatments. Values are means ± SD from three replicates. Columns with similar alphabets are not significantly different (*p* < 0.05).

**Figure 6:**
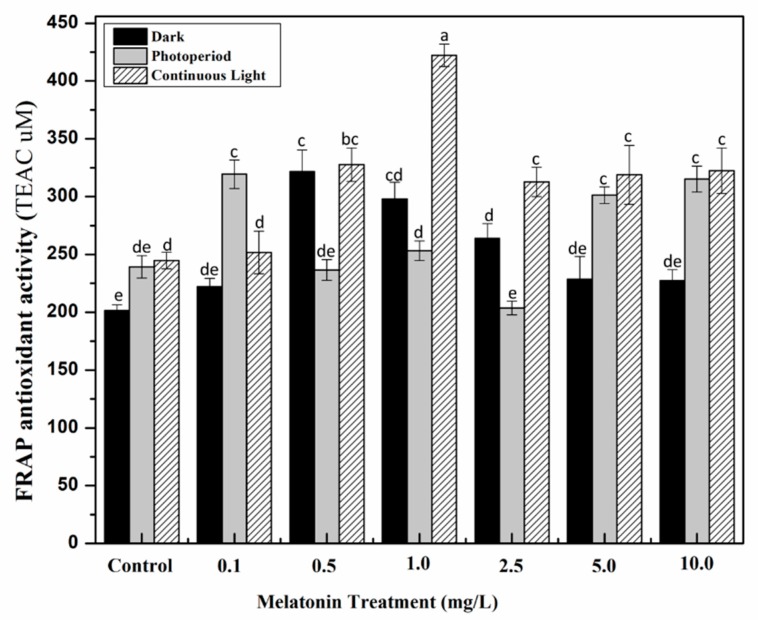
In vitro FRAP (ferric reducing antioxidant power) antioxidant activity of *S. marianum* calli grown under different light regimes and melatonin treatments. Values are means ± SD from three replicates. Columns with similar alphabets are not significantly different (*p* < 0.05).

Environmental stress on plants causes sudden shifts in their metabolic pathways which results in the production of reactive oxygen species that could damage plant cells, membrane lipids, proteins and DNA [49,50,51]. In response to oxidative stress, plants produce variety of metabolic compounds including phenolics, terpenoids and flavonoids that act as protecting mechanism [52,53,54]. Here, the antioxidant potential of *S. marianum* calli in response to different melatonin treatments and light regimes was also explored by employing three distinct antioxidant assays i.e., ABTS (2,2-azinobis-3-ethylbenzthiazoline-6-sulphonic acid) assay (hydrogen atom transfer (HAT)-based antioxidant assay), FRAP (ferric reducing antioxidant power) assay (electron transfer (ET)-based antioxidant activity) and DPPH (2,2-diphenyl-1-picrylhydrazyl) assay (mixt HAT- and ET-based antioxidant assay). Because of its mixed HAT- and ET-based antioxidant mechanism, DPPH quenching free radical activity was indicated as percentage (%) of free radical scavenging activity, whereas the ABTS and FRAP activities were demonstrated as TEAC (trolox C equivalent antioxidant capacity, μM). Highest DPPH activity (94.6%) was noted for cultures grown under continuous light with 1.0 mg/L melatonin, as compared to non-melatonin control (86.33%). Under dark conditions, 0.5 mg/L melatonin showed optimum scavenging activity (91.5%) (Figure 4). A similar trend was noted for FRAP and ABTS assays in which 1.0 mg/L melatonin treated cultures exhibited maximum FRAP (422.17 μM) and ABTS (771.48 μM) activities under continuous light, whereas, in the dark conditions, 0.5 mg/L melatonin resulted in optimum FRAP (321 μM) and ABTS (545.67 μM) activities (Figure 5 and Figure 6). Results of antioxidant activities revealed an obvious correlation with plant secondary metabolites. The synergistic role of continuous light and melatonin significantly increased phytochemical accumulation in callus cultures of *S. marianum* which subsequently enhanced its antioxidant potential. The highest antioxidant activity could be due to an increase in silymarin. Several studies have highlighted the potential role of silymarin in decreased production of reactive oxygen species by scavenging free radicals [55,56,57]. Similar findings showing the correlation of phenolic profiling with antioxidant potential have been reported in a wide variety of other plant species [58,59].

### 2.4. Effect of Light and Melatonin on Anti-Inflammatory Potential of S. Marianum Callus Cultures 

Inflammation is immune system’s response to pathogens, harmful stimuli, irritants and damaged cells. The in vitro and in vivo anti-inflammatory activities have been reported for several flavonoids. These flavonoids exert in vivo anti-inflammatory action through variety of mechanisms such as inhibition of cyclooxygenases with sometime a differential action on COX-1 vs. COX-2, phospholipase A2 and lipoxygenases (eicosanoid generating enzymes), thereby decreasing leukotrienes and prostanoid concentrations [60]. Different in vitro assays like COX-1, COX-2, 15-LOX and sPLA2 were here carried out to verify the anti-inflammatory potential of *S. marianum* callus cultures. Of all the treatments, continuous light with 1.0 mg/L melatonin gave most effective results towards the inhibitory actions of all assays performed. Highest inhibitory activity was shown against 15-LOX (42.33 ± 1.59%) followed by COX-1 (37.15 ± 1.29%), sPLA2 (35.70 ± 0.99%) and COX-2 (29.03 ± 0.97%), respectively in cultures grown under continuous light with 1.0 mg/L melatonin. Percent (%) inhibitions of different samples have been depicted in Table 2. Dark grown cultures showed maximum anti-inflammatory activity at 0.5 mg/L melatonin, whereas, cultures placed under continuous light displayed optimum results at 1.0 mg/L melatonin concentration. It has previously been established by many studies that silymarin contents are responsible for enhanced anti-inflammatory effect in *S. marianum* [61,62,63]. Pradhan et al. [64] also concluded that increased production of silymarin content significantly enhanced the anti-inflammatory activity. Phytochemicals in plants are solely responsible for enzymatic inhibition that causes inflammation in the body [19,65,66].

### 2.5. Effect of Light and Melatonin on Silymarin 

**Figure 7:**
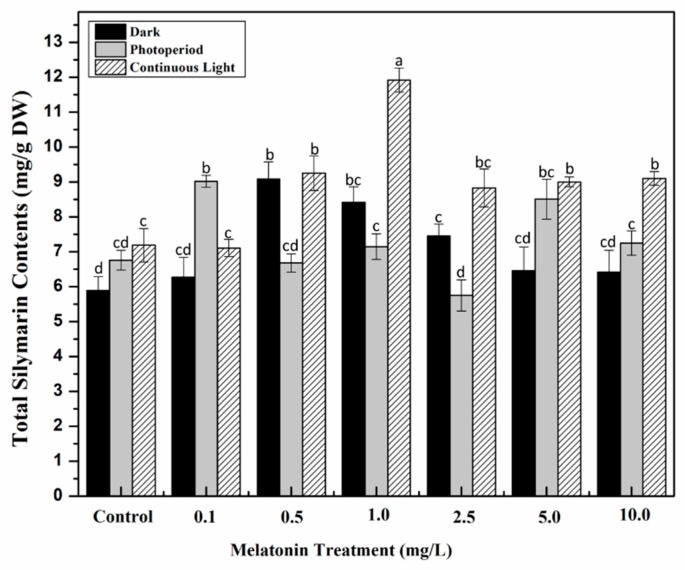
Total silymarin contents in callus cultures of *S. marianum* grown under different light regimes and melatonin treatments. Values are means ± SD from three replicates. Columns with similar alphabets are not significantly different (*p* < 0.05).

The individual composition of silymarin was also quantified in the current study against various melatonin concentrations and light regimes. Continuous light showed a prominent effect on total silymarin content (TSC) as compared to the rest of light regimes, whereas melatonin produced variable results in response to light treatment. Under continuous light, optimal TSC (11.92 mg/g DW) was noted in 1.0 mg/L melatonin treated cultures compared to control (7.189 mg/g DW). Similarly, maximum TSC (9.08 mg/g DW) was found in cultures grown in the dark with 0.5 mg/L melatonin. Cultures grown under photoperiod displayed highest TSC (9.01 mg/g DW) at 0.1 mg/L melatonin concentration (Figure 7). Results suggested that lower concentrations of melatonin favor optimum secondary metabolites biosynthesis by overcoming stress induced by continuous light and dark treatments. Our results are in harmony with those shown by Fazal et al. [39] who concluded that low exogenously applied melatonin concentration produced higher biomass accumulation and enzymatic activity in *P. vulgaris* cultures grown in vitro. Similarly, Adil at al. [41] also showed maximum secondary metabolites accumulation in melatonin treated cultures in continuous light as compared to dark and photoperiod regimes. High-performance liquid chromatography (HPLC) analysis of individual compounds showed that silybin A, silychristin and silybin B are the major compounds synthesized in callus cultures of Milk thistle, as previously reported in various studies [67,68]. Melatonin (1.0 mg/L) showed maximum accumulation of silybin A (1.45 mg/g DW), silychristin (1.08 mg/g DW) and silybin B (7.43 mg/g DW) under continuous light as compared to other treatments (Table 3). Silybin (A and B) are considered as primary compounds in Milk thistle extract as previously reported [67,69], which is in accordance with our study. Continuous light has a profound effect on biological synthesis of precious secondary metabolites. Previous studies on *O. basilicum* also revealed highest phytochemical accumulation grown under white light [70,71]. Taxifolin accumulation was also determined under different light regimes and melatonin concentrations. As compared to control (51.7 μg/g DW), maximum taxifolin (136 μg/g DW) was recorded for 1.0 mg/L melatonin treated cultures grown under continuous light. Younas et al. [72] also reported maximum taxifolin accumulation in *S. marianum* calli grown under continuous white light. Since silymarin contents are usually synthesized and derived from taxifolin in *S. marianum*, it is safe to assume that low level of taxifolin could be due to its conversion into silymarin contents [73,74].

## 3. Materials and Methods

### 3.1. Chemicals

All the chemicals used in the present study were of analytical grade quality and purchased from Thermo (Illkirch, France). The deionized water was produced using a milli-Q water purification system (Merck Millipore, Molsheim, France). Prior to their use for analysis, all solutions were filtered through 0.45 µm nylon syringe membranes (Merck Millipore, Molsheim, France). All phytohormones and commercial standards were purchased from Sigma-Aldrich (Saint-Quentin Fallavier, France). 

### 3.2. Seed Germination and Explant Collection

The seeds of *Silybum marianum* were taken from the seed bank of the Plant Cell Culture Lab (PCCL), Department of Biotechnology, Quaid-i-Azam University, Pakistan. Seeds were thoroughly washed and then subjected to surface sterilization. Mercuric chloride solution (0.1%) was used for 40 s, followed by ethanol washing (70%) for 90 seconds. Seeds were then washed again three times with autoclaved distilled water to free them from any unwanted particles. Surface sterilized seeds were then inoculated on Murashige and Skoog (MS) media (Murashige and Skoog 1962) [75] supplemented with agar (0.8%) and sucrose (3%) and placed in growth room with 25 ± 2 °C temperature and 16/8 h light/dark cycle (photoperiod). The media pH was maintained at 5.6–5.7, prior to being autoclaved at 121 °C for 20 min. Plantlets (4 weeks old) were then employed as a source of explant collection for callus induction.

### 3.3. Callus Culture Establishment

Leaf explants (0.5 cm^2^) were excised from in vitro germinated plantlets (4 weeks old) and placed on MS media containing different hormonal concentrations (0.5–10 mg/L) of thidiazuron (TDZ), 6-benzyl aminopurine (BAP) and α-naphthalene acetic acid (NAA), either alone or in conjunction with 1.0 mg/L NAA along with agar (0.8%) and sucrose (3%) for callus culture establishment. Callus culture was established under controlled environmental conditions in growth chamber. Four weeks old calli were then sub-cultured on respective hormonal media for maximum biomass production. 

### 3.4. Melatonin and Light Treatment

Preliminary results of callus culture optimization on different hormones showed optimum response on combined treatment of 0.5 mg/L BAP and 1.0 mg/L NAA (unpublished data), as compared to the rest of treatments. Four weeks old sub-cultured calli (1.0 g) at optimized hormonal concentration (0.5 mg/L BAP + 1.0 mg/L NAA) was then used to inoculate with various concentrations (0.1, 0.5, 1.0, 2.5, 5.0, 10 mg/L) of melatonin on the same optimum hormonal media. Callus without melatonin treatment was used as control. The whole experiment was treated with three different light regimes: dark (24 h), photoperiod (16/8 h light/dark) and continuous white light (24 h) at 25 ± 2 °C to check the interlinking effect of light and melatonin. Experiment was conducted in triplicate and harvested after 28 days for estimation of fresh weight (FW), dry weight (DW) and phytochemical contents. 

### 3.5. Total Flavonoid and Phenolic Contents

To investigate the accumulation of phytochemicals, extraction was carried out from dried samples according to Zahir et al. [56] with modifications. Dried powder (100 mg) from each sample was mixed with 99.9% methanol (500 µL) and vortexed for approximately 5 min, followed by sonication at room temperature for 30 min. Centrifugation of the reaction mixture was undertaken for 10 min at 15,000 rpm and the resultant supernatant was separately kept at 4 °C for phytochemical assays. Total phenolic content (TPC) was estimated with the help of a Folin–Ciocalteu (FC) reagent using slightly modified method of Singleton and Rossi (1965) [76]. Reaction mixture was prepared using methanol extracted sample (20 µL), FC reagent (90 µL) and NA_2_CO_3_ (90 µL). Gallic acid was employed as a standard and the TPC was expressed as gallic acid equivalents (GAE)/g of DW. Using microplate reader (BioTek ELX800 Absorbance Microplate Reader, BioTek Instruments, Colmar, France), the absorbance was taken at 630 nm. Similarly, total flavonoid content (TFC) was also measured using previously described aluminum chloride colorimetric method [77] with slight changes. Reaction mixture was prepared using the sample (20 µL), aluminum chloride (10 µL), distilled water (160 µL) and potassium acetate (10 µL) to make 200 µL of final volume. The standard used in this assay was quercetin and the TFC was expressed as quercetin equivalents (QE)/g of DW. Prior to noting absorbance at 415 nm using microplate reader (BioTek ELX800 Absorbance Microplate Reader, BioTek Instruments, Colmar, France), the reaction mixture was incubated for half an hour. 

### 3.6. Estimation of Antioxidant Activity

#### 3.6.1. DPPH Activity (%)

DPPH (2,2-diphenyl-1-picrylhydrazyl) quenching free radical activity of the samples was performed using the protocol of Abbasi et al. [78]. 20 μL sample was added into each well of microplate, followed by addition of DPPH reagent solution of 180 μL and then incubated in the dark at room temperature for 60 min. Ascorbic acid final concentrations (05, 10, 20 and 40 μg/mL) and 180 μL of DPPH with 20 μL of DMSO were taken as negative control. Microplate reader (BioTek ELX800 Absorbance Microplate Reader, BioTek Instruments, Colmar, France) was used to record the solution absorbance at 517 nm. The following formula was then employed to calculate DPPH activity:

% scavenging = 100 × (1 − AE/AD)(1)

where, AE = absorbance of the mixture at 517 nm with addition of sample, and AD = absorbance of DPPH solution without addition of anything.

#### 3.6.2. Ferric Reducing Antioxidant Power (FRAP) Assay

For this assay, previous method of Benzie and Strain was followed [79]. Briefly, 190 μL of FRAP solution [containing 2,4,6-Tri(2-pyridyl)-s-triazine (TPTZ; 10 mM); acetate buffer (300 mM) of pH 3.6 and 20 mM ferric chloride hexahydrate (FeCl3.6H2O); ratio 10:1:1 (*v*/*v*/*v*)] was mixed with 10 μL of samples. The reaction mixtures were then put for 15 min at room temperature. Using microplate reader (BioTek ELX800 Absorbance Microplate Reader, BioTek Instruments, Colmar, France), the absorbance was noted at 630 nm. Tests were carried out in triplicates and the antioxidant activity was demonstrated as TEAC.

#### 3.6.3. Antioxidant ABTS Assay

Procedure described by Velioglu et al. [80] was employed for the ABTS (2,2-azinobis-3-ethylbenzthiazoline-6-sulphonic acid) assay. Briefly, the ABTS solution was prepared by mixing 7 mM ABTS salt equal proportion with 2.45 mM potassium per sulphate and the same mixture was then put in the dark for 16 h. The solution absorbance was noted at 734 nm and, before mixing with extracts, it was adjusted to 0.7. The mixture was put again in the dark for 15 min at room temperature (25 ± 1 °C) and the absorbance was then noted at 734 nm with the aid of a microplate reader (BioTek ELX800 Absorbance Microplate Reader, BioTek Instruments, Colmar, France). The assays were performed in triplicates and the antioxidant activity was expressed as TEAC. 

### 3.7. Anti-Inflammatory Activities

#### 3.7.1. Inhibitory Activity Against COX-1 and COX-2 

The inhibitory activity of selected samples were checked against COX-2 and COX-1 using COX-2 (human) and COX-1 (Ovine) assay kit (701050, Cayman Chem. Co, Interchim, Montluçon, France) following the instructions of the manufacturer. The substrate at 1.1 mM concentration was arachidonic acid and the positive control was ibuprofen (10 µM). The COXs peroxidase component was measured by the kit. The Synergy II reader (BioTek Instruments, Colmar, France) was used at 590 nm in a 96-well microplate for 5 min to check oxidized *N*,*N*,*N*′,*N*′-tetramethyl-*p*-phenylenediamine. 

#### 3.7.2. Inhibitory Activity Against 15-LOX 

To check the inhibitory activity of the samples against 15-LOX, assay kit (760700, Cayman Chem. Co., Interchim, Montluçon, France) was used following the instructions of the manufacturer. The substrate taken was arachidonic acid (10 µM) while 100 µM nordihydroguaiaretic acid (NDGA) was taken as positive control inhibitor. The hydroperoxides concentration produced during the lipooxygenation reaction was measured by the kit using filtered soy 15-lipooxygenase standard in 10 mM Tris-HCl buffer at 7.4 pH. Synergy II reader (BioTek Instruments, Colmar, France) was used for measurement at 940 nm in a 96-well microplate. After 5 min incubation of the inhibitor and enzyme, the absorbance was recorded followed by incubation of 15 min after addition of the substrate and incubation for 5 min after addition of chromogen.

#### 3.7.3. Inhibitory Activity Against Secretory Phospholipase A2 (sPLA2)

To test the inhibitory ability of samples against sPLA2, an assay kit (10004883, Cayman Chem. Co, Interchim, Montluçon, France) was used following instructions of the manufacturer. The substrate was diheptanoyl thio-PC (1.44 mM) while the thiotheramide-PC (100 µM) was used as a positive control inhibitor. The cleavage of diheptanoyl thio-PC ester releases free thiols which was measured by Synergy II reader (BioTek Instruments, Colmar, France) at 420 nm in a 96-well microplate using DTNB (5-5′-dithio-bis-(2-nitrobenzoic acid). The % inhibition was calculated as:

% Inhibition = [(IA − Inhibitor)/IA] × 100(2)

where, Inhibitor = Activity of enzyme with inhibitor addition; IA = 100% activity of enzyme in the absence of inhibitor.

### 3.8. High-Performance Liquid Chromatography Electrospray Ionization Mass Spectrometry (HPLC-ESI-MS) Analysis

Liquid chromatography-mass spectrometry (LC-MS) analysis was done to quantify silymarin compounds, as previously described by Drouet et al. [81] using using aWaters 2695 Alliance coupled with a single quadrupole mass spectrometer ZQ (Waters-Micromass, Manchester, UK), equipped with an electrospray ion source (ESI-MS). The examination of all samples was done three times, and the results were revealed as mg/g sample DW. Taxofolin and flavonolignans were identified by comparison with authentic standards (Sigma Aldrich). The linear correlations between peak area and standard concentrations were found to be high in the range of 0.5–50 µg/mL. The resulting linear equations were obtained with R^2^-value for a six-point calibration graph > 0.99, with Taxifolin (y = 1292.9 + 0.7, R^2^ = 0.9989, limit of detection (LOD) = 0.09 µg/mL, limit of quantitation (LOQ) = 0.26 µg/mL), silychristin (y = 2266.4 – 12.7, R^2^ = 0.9994, LOD = 0.05 µg/mL, LOQ = 0.17 µg/mL), silydianin (y = 1649.8 + 34.8, R^2^ = 0.9992, LOD = 0.10 µg/mL, LOQ = 0.32 µg/mL), silybin A (y = 2575.0 + 5.7, R^2^ = 0.9997, LOD = 0.05 µg/mL, LOQ = 0.15 µg/mL), silybin B (y = 2515.9 + 27.3, R^2^=0.9999, LOD = 0.05 µg/mL, LOQ = 0.15 µg/mL), isosilybin A (y = 2726.3 + 17.8, R^2^ = 0.9998, LOD = 0.05 µg/mL, LOQ = 0.16 µg/mL), isosilybin B (y = 2861.1 + 1.9, R^2^ = 0.9999, LOD = 0.05 µg/mL, LOQ = 0.16 µg/mL).

### 3.9. Statistical Analysis

All of the experiments were carried out in an organized manner, with each treatment examined thrice (biological replicates), and repeated twice (technical replicates). Origin (Windows v8.5) software (OriginLab Corporation, Wellesley Hills, MA, USA) was employed for statistical analysis and analytical data was revealed as mean ± standard deviation with the help of Excel 2018 (Microsoft, Redmond, WA, USA).

## 4. Conclusions

In conclusion, melatonin with different light regimes strongly influences the biosynthesis of active ingredients in callus cultures of *S. marianum*. BAP and NAA in combination favors optimal callus induction from leaf explants. Moreover, continuous light (24 h) proved to be the most effective as an abiotic elicitor as compared to the rest of light regimes, whereas, higher melatonin concentrations have inhibitory effect on biomass accumulation and phytochemical biosynthesis. Additionally, continuous light with 1.0 mg/L melatonin is the best for enhanced total silymarin content (11.92 mg/g DW) along with increased anti-inflammatory and antioxidant properties in *S. marianum* callus cultures, thus making silymarin clinically more attractive for these properties. Previously, the anti-inflammatory effect and antioxidant potential of silymarin in Type 2 diabetes patients have been demonstrated [82]. Similarly, silymarin has been reported to decrease the elevated levels of complement proteins and interleukins in patients with knee osteoarthritis, when used either alone or in conjunction with non-steroidal anti-inflammatory drugs [83]. The results of our study indicate that silybin (A and B) and silychristin are the major secondary metabolites produced in *S. marianum* callus cultures. Several experimental reports have indicated the metabolic, antioxidant and antifibrotic effects of silybin. Although earlier human studies lack adequate confirmation towards the clinical efficacy of silybin in chronic liver diseases, silybin seems to be a promising candidate in the management of chronic liver diseases on the basis of available literature and ongoing clinical trials [84]. Traditional cultivation of milk thistle plants is prone to many complications which results in reduced total yield, mainly due to the spiny nature of the flowers and leaves. Furthermore, using herbicides leads to contamination of the seeds (fruits) with toxins. To overcome these complications, an in vitro propagation system is extremely useful. This study helps to better comprehend the influence of melatonin and different light regimes on secondary metabolites production as well as antioxidant and anti-inflammatory activities in in vitro callus cultures of *S. marianum* and paves the way for the use of this production system for future nutraceutical or cosmeceutical applications. However, techniques like high-throughput sequencing are still required in order to elucidate the effect of melatonin and different light regimes on metabolic pathways underlying silymarin biosynthesis in *S. marianum*.

## Figures and Tables

**Table 1:** Interactive effect of different light regimes and melatonin treatments on biomass production in callus culture of *S. marianum*.

Treatment	Dark (24 h)		Photoperiod (16 h/8 h)		Continuous Light (24 h)	
Melatonin (mg/L)	FW (g/L)	DW (g/L)	FW (g/L)	DW (g/L)	FW (g/L)	DW (g/L)
**Control**	170.47 ± 3.83 ^**d**^	10.74 ± 0.38 ^**bc**^	188.9 ± 5.38 ^**cd**^	11.27 ± 0.64 ^**bc**^	215.4 ± 4.07 ^**c**^	12.56 ± 0.50 ^**b**^
**0.1**	210.888 ± 4.09 ^**c**^	12.36 ± 0.25 ^**b**^	149.8 ± 4.30 ^**de**^	12.78 ± 0.53 ^**b**^	252.6 ± 6.42 ^**b**^	13.93 ± 0.57 ^**ab**^
**0.5**	159.9 ± 5.20 ^**de**^	13.11 ± 1.37 ^**ab**^	177.14 ± 4.86 ^**d**^	14.37 ± 0.13 ^**ab**^	266.7 ± 7.6 ^**ab**^	14.29 ± 0.83 ^**ab**^
**1.0**	135.78 ± 7.78 ^**e**^	10.79 ± 0.90 ^**bc**^	205.88 ± 3.42 ^**c**^	13.87 ± 0.92 ^**ab**^	299.3 ± 6.57 ^**a**^	15.96 ± 0.68 ^**a**^
**2.5**	131.58 ± 6.03 ^**e**^	10.66 ± 0.49 ^**bc**^	175.01 ± 4.46 ^**d**^	10.39 ± 0.50 ^**bc**^	204.3 ± 9.72 ^**c**^	12.57 ± 0.37 ^**b**^
**5.0**	101.44 ± 4.75 ^**ef**^	8.26 ± 0.50 ^**c**^	165.94 ± 5.92 ^**d**^	12.33 ± 0.35 ^**b**^	215.5 ± 8.28 ^**c**^	13.10 ± 1.01 ^**ab**^
**10.0**	93.46 ± 3.85 ^**f**^	7.54 ± 0.46 ^**cd**^	152.6 ± 5.73 ^**de**^	11.65 ± 0.42 ^**b**^	260.5 ± 6.8 ^**ab**^	14.73 ± 1.34 ^**a**^

Values are means ± SD from triplicates. Columns with similar alphabets are not significantly different (*p* < 0.05). (FW = fresh weight; DW = dry weight).

**Table 2:** Anti-inflammatory potential of *S. marianum* callus cultures grown under different light regimes and melatonin treatments.

Light Regime	Melatonin (mg/L)	15-LOX (% Inh)	COX-1 (% Inh)	sPLA_2_ (% Inh)	COX-2 (% Inh)
**Dark**	**Control**	18.56 ± 1.05 ^**c**^	20.55 ± 1.08 ^**cd**^	19.83 ± 1.45 ^**bc**^	13.37 ± 1.34 ^**bc**^
	**0.1**	20.90 ± 1.43 ^**c**^	21.20 ± 1.30 ^**cd**^	22.27 ± 0.95 ^**bc**^	15.93 ± 2.01 ^**bc**^
	**0.5**	**30.99 ± 0.99 ^**b**^**	**28.60 ± 1.54 ^**b**^**	**28.96 ± 1.03 ^**ab**^**	**22.24 ± 1.34 ^**ab**^**
	**1.0**	28.74 ± 2.56 ^**b**^	26.94 ± 0.83 ^**bc**^	27.37 ± 1.30 ^**ab**^	20.78 ± 0.94 ^**b**^
	**2.5**	26.41 ± 1.98 ^**bc**^	25.51 ± 1.54 ^**bc**^	25.08 ± 1.28 ^**b**^	19.05 ± 1.56 ^**b**^
	**5.0**	21.38 ± 2.03 ^**c**^	21.47 ± 1.48 ^**cd**^	22.70 ± 1.84 ^**bc**^	16.25 ± 2.01 ^**bc**^
	**10.0**	20.99 ± 3.51 ^**c**^	21.07 ± 1.56 ^**cd**^	22.62 ± 1.26 ^**bc**^	16.05 ± 1.67 ^**bc**^
**Photoperiod**	**Control**	21.87 ± 2.50 ^**c**^	21.62 ± 1.62 ^**cd**^	23.42 ± 1.49 ^**bc**^	16.68 ± 0.93 ^**bc**^
	**0.1**	**30.13 ± 1.87 ^**b**^**	**27.90 ± 1. 38 ^**b**^**	**28.80 ± 0.84 ^**ab**^**	**21.89 ± 1.45 ^**ab**^**
	**0.5**	22.59 ± 1.14 ^**bc**^	22.48 ± 2.05 ^**c**^	23.23 ± 2.03 ^**b**^	16.92 ± 1.04 ^**bc**^
	**1.0**	24.07 ± 1.41 ^**bc**^	23.47 ± 1.56 ^**c**^	24.35 ± 1.82 ^**b**^	17.88 ± 0.72 ^**bc**^
	**2.5**	18.72 ± 1.78 ^**c**^	19.51 ± 0.83 ^**cd**^	21.02 ± 0.59 ^**bc**^	14.63 ± 1.94 ^**bc**^
	**5.0**	28.45 ± 1.05 ^**b**^	26.64 ± 1.55 ^**bc**^	27.58 ± 1.47 ^**ab**^	20.77 ± 2.04 ^**b**^
	**10.0**	26.36 ± 1.32 ^**bc**^	24.66 ± 1.83 ^**c**^	22.98 ± 2.44 ^**bc**^	18.66 ± 1.43 ^**b**^
**Continuous Light**	**Control**	28.37 ± 1.69 ^**b**^	22.66 ± 0.80 ^**c**^	21.79 ± 1.28 ^**bc**^	16.23 ± 1.93 ^**bc**^
	**0.1**	23.65 ± 0.97 ^**bc**^	23.09 ± 0.92 ^**c**^	24.25 ± 1.66 ^**b**^	17.68 ± 1.03 ^**bc**^
	**0.5**	31.49 ± 1.23 ^**b**^	28.97 ± 1.56 ^**b**^	29.35 ± 1.22 ^**ab**^	22.59 ± 1.05 ^**ab**^
	**1.0**	**42.33 ± 1.59 ^**a**^**	**37.15 ± 1.29 ^**a**^**	**35.70 ± 0.99 ^**a**^**	**29.03 ± 0.97 ^**a**^**
	**2.5**	29.95 ± 1.86 ^**b**^	27.80 ± 1.45 ^**b**^	28.35 ± 2.76 ^**ab**^	21.62 ± 1.46 ^**ab**^
	**5.0**	30.87 ± 1.23 ^**b**^	28.52 ± 1.33 ^**b**^	28.76 ± 1.55 ^**ab**^	22.11 ± 1.35 ^**ab**^
	**10.0**	30.82 ± 1.50 ^**b**^	28.45 ± 1.20 ^**b**^	29.00 ± 1.39 ^**ab**^	22.20 ± 1.04 ^**ab**^

15-LOX: Arachidonate 15-Lipoxygenase; sPLA_2_: phospholipase A2; COX: cyclooxygenase. Values are means ± SD from three replicates. Columns with similar alphabets are not significantly different (*p* < 0.05). (% inh = percent inhibition). The highest inhibition percentages are in bold.

**Table 3:** Quantification of silymarin compounds in callus cultures of *S. marianum* grown under different light regimes and melatonin treatments.

Light Regimes	Melatonin (mg/L)	Silymarin Compounds (mg/g DW)							
		Silybin A	Silybin B	Isosilybin A	Isosilybin B	Silychristin	Isosilychristin	Silydianin	Taxifolin
**Dark**	**Control**	0.62 ± 0.05 ^**cd**^	3.34 ± 0.04 ^**e**^	0.17 ± 0.002 ^**b**^	0.15 ± 0.03 ^**bc**^	0.58 ± 0.06 ^**c**^	0.28 ± 0.02 ^**bc**^	0.60 ± 0.08 ^**bc**^	0.05 ± 0.009 ^**b**^
	**0.1**	0.67 ± 0.002 ^**cd**^	3.67 ± 0.06 ^**de**^	0.19 ± 0.04 ^**ab**^	0.15 ± 0.02 ^**bc**^	0.60 ± 0.03 ^**c**^	0.29 ± 0.09 ^**bc**^	0.61 ± 0.064 ^**b**^	0.06 ± 0.005 ^**b**^
	**0.5**	1.06 ± 0.045 ^**b**^	5.59 ± 0.076 ^**bc**^	0.22 ± 0.055 ^**a**^	0.22 ± 0.054 ^**ab**^	0.82 ± 0.028 ^**b**^	0.38 ± 0.059 ^**ab**^	0.68 ± 0.055 ^**ab**^	0.13 ± 0.003 ^**a**^
	**1.0**	0.97 ± 0.037 ^**bc**^	5.13 ± 0.056 ^**c**^	0.21 ± 0.029 ^**ab**^	0.21 ± 0.053 ^**b**^	0.77 ± 0.048 ^**bc**^	0.36 ± 0.040 ^**b**^	0.66 ± 0.087 ^**b**^	0.08 ± 0.006 ^**ab**^
	**2.5**	0.82 ± 0.039 ^**c**^	4.37 ± 0.042 ^**d**^	0.21 ± 0.019 ^**ab**^	0.19 ± 0.048 ^**b**^	0.73 ± 0.098 ^**bc**^	0.33 ± 0.076 ^**ab**^	0.67 ± 0.067 ^**ab**^	0.08 ± 0.004 ^**ab**^
	**5.0**	0.70 ± 0.048 ^**cd**^	3.81 ± 0.039 ^**de**^	0.19 ± 0.074 ^**ab**^	0.16 ± 0.047 ^**bc**^	0.60 ± 0.038 ^**c**^	0.29 ± 0.033 ^**bc**^	0.61 ± 0.062 ^**b**^	0.06 ± 0.007 ^**b**^
	**10.0**	0.69 ± 0.044 ^**cd**^	3.82 ± 0.027 ^**de**^	0.19 ± 0.06 ^**ab**^	0.15 ± 0.051 ^**bc**^	0.59 ± 0.064 ^**c**^	0.29 ± 0.009 ^**bc**^	0.60 ± 0.094 ^**bc**^	0.06 ± 0.008 ^**b**^
**Photoperiod**	**Control**	0.74 ± 0.04 ^**c**^	4.08 ± 0.03 ^**d**^	0.19 ± 0.05 ^**ab**^	0.16 ± 0.02 ^**bc**^	0.60 ± 0.07 ^**c**^	0.30 ± 0.04 ^**bc**^	0.59 ± 0.09 ^**bc**^	0.06 ± 0.004 ^**b**^
	**0.1**	1.05 ± 0.09 ^**b**^	5.59 ± 0.89 ^**bc**^	0.21 ± 0.04 ^**ab**^	0.21 ± 0.04 ^**b**^	0.79 ± 0.05 ^**b**^	0.37 ± 0.06 ^**ab**^	0.66 ± 0.01 ^**b**^	0.09 ± 0.002 ^**ab**^
	**0.5**	0.72 ± 0.06 ^**c**^	3.93 ± 0.74 ^**de**^	0.21 ± 0.05 ^**ab**^	0.16 ± 0.06 ^**bc**^	0.64 ± 0.09 ^**c**^	0.30 ± 0.08 ^**bc**^	0.63 ± 0.05 ^**b**^	0.06 ± 0.008 ^**b**^
	**1.0**	0.79 ± 0.048 ^**c**^	4.27 ± 0.55 ^**d**^	0.20 ± 0.07 ^**ab**^	0.17 ± 0.08 ^**bc**^	0.66 ± 0.03 ^**c**^	0.31 ± 0.07 ^**b**^	0.63 ± 0.03 ^**b**^	0.07 ± 0.006 ^**b**^
	**2.5**	0.60 ± 0.07 ^**cd**^	3.34 ± 0.48 ^**e**^	0.18 ± 0.095 ^**b**^	0.14 ± 0.03 ^**bc**^	0.54 ± 0.02 ^**cd**^	0.27 ± 0.03 ^**bc**^	0.59 ± 0.08 ^**bc**^	0.05 ± 0.005 ^**b**^
	**5.0**	0.98 ± 0.039 ^**bc**^	5.23 ± 0.95 ^**c**^	0.19 ± 0.004 ^**ab**^	0.20 ± 0.05 ^**b**^	0.75 ± 0.06 ^**bc**^	0.36 ± 0.05 ^**b**^	0.65 ± 0.09 ^**b**^	0.08 ± 0.007 ^**ab**^
	**10.0**	0.81 ± 0.055 ^**c**^	4.34 ± 0.33 ^**d**^	0.20 ± 0.056 ^**ab**^	0.18 ± 0.07 ^**b**^	0.67 ± 0.04 ^**c**^	0.32 ± 0.01 ^**b**^	0.63 ± 0.05 ^**b**^	0.07 ± 0.003 ^**b**^
**Continuous Light**	**Control**	0.80 ± 0.05 ^**c**^	4.32 ± 0.98 ^**d**^	0.20 ± 0.03 ^**ab**^	0.17 ± 0.01 ^**bc**^	0.66 ± 0.02 ^**c**^	0.31 ± 0.07 ^**b**^	0.63 ± 0.04 ^**b**^	0.051 ± 0.005 ^**b**^
	**0.1**	0.79 ± 0.03 ^**c**^	4.27 ± 0.07 ^**d**^	0.20 ± 0.044 ^**ab**^	0.17 ± 0.03 ^**bc**^	0.65 ± 0.06 ^**c**^	0.31 ± 0.09 ^**b**^	0.62 ± 0.08 ^**b**^	0.07 ± 0.007 ^**b**^
	**0.5**	1.08 ± 0.07 ^**b**^	5.71 ± 0.05 ^**bc**^	0.22 ± 0.096 ^**a**^	0.22 ± 0.07 ^**b**^	0.83 ± 0.05 ^**b**^	0.38 ± 0.03 ^**ab**^	0.68 ± 0.06 ^**ab**^	0.09 ± 0.009 ^**ab**^
	**1.0**	**1.45 ± 0.05^**a**^**	**7.43 ± 0.06 ^**a**^**	**0.24 ± 0.055 ^**a**^**	**0.30 ± 0.05 ^**a**^**	**1.08 ± 0.08 ^**a**^**	**0.47 ± 0.04 ^**a**^**	**0.77 ± 0.01 ^**b**^**	**0.13 ± 0.005 ^**a**^**
	**2.5**	1.03 ± 0.08 ^**b**^	5.42 ± 0.04 ^**c**^	0.21 ± 0.039 ^**ab**^	0.21 ± 0.08 ^**b**^	0.79 ± 0.03 ^**b**^	0.37 ± 0.06 ^**ab**^	0.66 ± 0.05 ^**b**^	0.09 ± 0.008 ^**ab**^
	**5.0**	1.05 ± 0.06 ^**b**^	5.52 ± 0.098 ^**c**^	0.22 ± 0.084 ^**a**^	0.22 ± 0.04 ^**b**^	0.82 ± 0.09 ^**b**^	0.37 ± 0.032 ^**ab**^	0.68 ± 0.03 ^**ab**^	0.09 ± 0.004 ^**ab**^
	**10.0**	1.06 ± 0.04 ^**b**^	5.62 ± 0.05 ^**bc**^	0.22 ± 0.044 ^**a**^	0.22 ± 0.02 ^**b**^	0.81 ± 0.04 ^**b**^	0.38 ± 0.085 ^**ab**^	0.67 ± 0.07 ^**ab**^	0.08 ± 0.003 ^**ab**^

Values are means ± SD from three replicates. Columns with similar alphabets are not significantly different (*p* < 0.05). (Optimum values are highlighted bold).
